# Preoperative Anxiety and Information Desire Among Patients Undergoing Elective Surgery in Northern Sudan: Multicenter Cross-Sectional Study

**DOI:** 10.2196/75736

**Published:** 2025-10-15

**Authors:** Abeer Ahmed, Mohamed Nasur, Eman Mohamed, Amna Faragalla, Mustafa Ahmed, Murouj Mohammed, Abdinur Yusuf, Mohamed Issak

**Affiliations:** 1 Department of Public Health, Postgraduate School, University of Dongola, Dongola, Sudan; 2 Faculty of Medicine and Health Science, University of Dongola, Faculty of Medicine Street, Dongola, 11111, Sudan, 249 999665700; 3 Faculty of Medicine, University of Gezira, Wad Madani, Sudan; 4 Department of Internal Medicine, Al-Wifaq Al-Qatari Hospital, Al Dabbah, Sudan; 5 Faculty of Medicine, Merowe University of Technology, Merowe, Sudan; 6 Internal Medicine Council, Sudan Medical Specialization Board, Atbara, Sudan

**Keywords:** preoperative anxiety, desire for information, risk factors, elective surgery, Sudan

## Abstract

**Background:**

Preoperative anxiety is a common psychological condition, and many patients express a desire for more information before surgery. Understanding the prevalence and associated factors of both preoperative anxiety and the desire for information can improve patient care.

**Objective:**

This study aimed to assess the prevalence of preoperative anxiety and desire for information, as well as their associated sociodemographic, medical, and surgical factors, among patients undergoing elective surgery in Northern State, Sudan.

**Methods:**

A hospital-based, multicenter, cross-sectional study was conducted from November 2024 to February 2025 in Northern State, Sudan, involving patients undergoing elective surgery. Data were collected through face-to-face interviews using a structured questionnaire and the validated Arabic version of the Amsterdam Preoperative Anxiety and Information Scale (APAIS). Chi-square tests and univariate and multivariate logistic regression were performed to identify the associated factors and the magnitude, with statistical significance set at *P*<.05.

**Results:**

Of the 380 patients approached, 305 participated in the study (response rate=80.3%): 173 of the 305 participants (56.7%) were male, and the median age was 43 (IQR 30‐64) years. Most participants were married (n=207, 67.9%), educated (n=248, 81.3%), and had family support (n=253, 83.0%). Regarding surgical characteristics, the majority underwent either intermediate (n=136, 44.6%) or major (n=142, 46.6%) procedures. General anesthesia was the most common type used (n=159, 52.2%), and most participants (n=169, 55.4%) underwent surgery in public hospitals. Most participants reported that their surgeries were not covered by insurance (n=264, 86.6%) and described good sleep quality the night before surgery (n=221, 72.5%). Of the 305 participants, 75 (24.6%) experienced preoperative anxiety, whereas 92 (30.1%) expressed a moderate to high desire for information. Preoperative anxiety was significantly associated with family support (adjusted odds ratio [aOR] 7.12, 95% CI 2.64‐19.23; *P*<.001), surgery in public hospitals (aOR 4.31, 95% CI 2.30‐8.07; *P*<.001), poor sleep quality the night before surgery (aOR 2.85, 95% CI 1.51‐5.38; *P*=.001), and American Society of Anesthesiologists (ASA) classification III/IV (aOR 2.36, 95% CI 1.00‐5.54; *P*=.049). Similarly, a higher desire for information was significantly associated with being educated (aOR 2.48, 95% CI 1.00‐6.11; *P*=.049), having family support (aOR 4.10, 95% CI 1.81‐9.30; *P*=.001), undergoing surgery in a public hospital (aOR 3.57, 95% CI 1.93‐6.61; *P*<.001), and being classified as ASA III/IV (aOR 3.26, 95% CI 1.39‐7.64; *P*=.001).

**Conclusions:**

Preoperative anxiety and desire for information are common among Sudanese patients. Family involvement may paradoxically increase anxiety and the desire for more information due to shared concerns and cultural factors. Other significant predictors of anxiety include poor sleep quality and higher ASA classification. Additionally, education, family support, and chronic diseases were associated with a higher desire for information. Addressing these factors may alleviate preoperative anxiety, satisfy communication needs, and improve preoperative care.

## Introduction

Anxiety is a subjective state of emotional unease, distress, or apprehension, often arising from the anticipation of danger and accompanied by autonomic and somatic symptoms that can impair functioning. While occasional anxiety is common, anxiety disorders involve intense and excessive fear and worry [1]. Approximately 33.7% of the global population experiences an anxiety disorder during their lifetime [2].

Anxiety becomes particularly concerning in medical contexts, such as surgery, where it can significantly impact outcomes and well-being [3]. Elective surgical procedures, which are nonemergency procedures that can be delayed for at least 24 hours, often trigger preoperative anxiety in patients [4]. This anxiety is characterized by worry, nervousness, and unease about the impending procedure and its potential outcomes [3,5].

Preoperative anxiety may have significant negative impacts on patients. It is associated with increased anesthesia requirements and hemodynamic instability during surgery as well as postoperative complications such as nausea, vomiting, and dizziness [6,7]. High anxiety levels are also linked to poor postoperative pain control, increased analgesic needs, delayed recovery, longer hospital stays, and complications such as surgical site infections and impaired wound healing [8-10]. These adverse effects may reduce patient satisfaction, hinder rehabilitation, and increase health care costs, making preoperative anxiety not only a psychological issue but also a clinical and economic one.

The global pooled prevalence of preoperative anxiety is estimated at 48% [11]. In low- and middle-income countries (LMICs), the issue is exacerbated by limited health care resources, inadequate patient information, and cultural beliefs surrounding surgery. Studies in LMICs reveal even higher prevalence rates, ranging from 55% to 99%, highlighting the need for context-specific research and interventions [12]. Neighboring countries such as Ethiopia and Yemen also report varying rates, influenced by factors such as surgery type, patient demographics, prior surgical experiences, and individual coping mechanisms [13,14].

In Sudan, where the health care system faces significant constraints due to the ongoing war, the issue of preoperative anxiety among patients undergoing elective surgery may be particularly pronounced. Factors such as limited health facilities, lack of access to information, long waiting times, and communication barriers may contribute to heightened anxiety levels. Moreover, the socioeconomic conditions and cultural beliefs of the Sudanese population may shape patients’ perceptions, expectations, and surgical experiences.

Despite growing global recognition of preoperative anxiety as a significant health concern, it remains underresearched in Sudan. Understanding this issue is essential for guiding health authorities and care providers in developing targeted interventions that reduce anxiety, enhance patient satisfaction, and improve surgical outcomes in resource-limited and conflict-affected settings such as Sudan.

Therefore, this pioneering study—the first of its kind in Sudan—aims to determine the prevalence of preoperative anxiety and desire for information and identify associated sociodemographic and clinical predictors among surgical patients in Northern Sudan.

## Methods

### Study Design

A multicenter, cross-sectional study was conducted over 4 months, from November 2024 to February 2025, in 4 hospitals with operation theaters in Northern State, Sudan: 2 public hospitals (Dongola Specialized Hospital and Karima Teaching Hospital) and 2 private hospitals (Al-Wifaq Al-Qatari Hospital and Al-Daman Hospital). The study followed the Strengthening the Reporting of Observational Studies in Epidemiology (STROBE) guidelines for observational studies [15].

### Study Population

The study included adult patients (≥18 years) undergoing elective surgery who provided written informed consent. Exclusion criteria were emergency or obstetric surgeries, a history of mental illness or cognitive impairment, and incomplete responses to the study questionnaire.

### Sample Size and Sampling Technique

The sample size was calculated using Cochran’s formula: sample size (n) = $\frac{z^{2}pq}{e^{2}}$ [16]. Using a prevalence of 55.7% (from similar LMIC studies), 95% confidence level (*z*=1.96), and 5% margin of error, the minimum sample size was determined to be 380 [7,16]. In total, 305 of the 380 distributed surveys were completed, yielding a response rate of 80.3%. Patients were recruited using convenience sampling from surgical wards and outpatient clinics 1-2 days before their scheduled operation.

### Data Collection

Data were collected through structured face-to-face interviews (within 48 h prior to the scheduled surgery) using a standardized questionnaire comprising 3 sections: a sociodemographic section with 7 items, a medical and surgical history section with 7 items, and the Amsterdam Preoperative Anxiety and Information Scale (APAIS) with 6 items .

The questionnaire was translated into Arabic, and the validated Arabic version of the APAIS was used [17]. A pilot test was conducted among 40 participants to ensure clarity and reliability, yielding a Cronbach α of 0.91, indicating excellent reliability [18]. The English version of the questionnaire is provided as Multimedia Appendix 1.

### Measurement of Preoperative Anxiety and Desire for Information

The APAIS was used to assess preoperative anxiety and desire for information. It is a validated scale with 6 items, each scored on a 5-point Likert scale ranging from 1 (“not at all”) to 5 (“extremely”). Preoperative anxiety was assessed through items 1, 2, 4, and 5, leading to a total score range of 4‐20, with a score ≥11 indicating clinically significant anxiety. Desire for information was assessed using items 3 and 6. The total scores ranged from 2 to 10 and were categorized as low (2-4), average (5-7), or high (8-10) desire for information [19-21].

### Data Analysis

#### Descriptive Analysis

SPSS (version 27; IBM Corp) was used for all statistical analyses. Descriptive statistics (frequencies and percentages) summarized the sociodemographic, medical, and surgical characteristics of participants, as well as the prevalence of preoperative anxiety and desire for information.

#### Bivariate Analysis

Chi-square tests were used to examine associations between independent variables (eg, sociodemographics and surgical details) and the outcomes: preoperative anxiety and desire for information. A *P* value of <.05 was considered statistically significant.

#### Multivariate Analysis

Variables with a *P* value <.05 in the bivariate analysis were included in a binary logistic regression model for preoperative anxiety and desire for information. Adjusted odds ratios (aORs) with 95% CI were calculated to determine independent predictors.

### Ethical Considerations

The study was conducted in accordance with the Declaration of Helsinki. Ethical approval was obtained from the Research Ethical Committee of the Northern State Ministry of Health (REC-MoH-NS-41‐2023) and the research offices of the involved hospitals. Written informed consent was obtained from all participants prior to their inclusion in the study. Participants were assured of the confidentiality of their responses, the anonymity of their data, and their right to withdraw from the study at any time without any impact on their medical care. No compensation was provided to participants for their involvement in the study.

## Results

### General Characteristics of the Study Population

**Figure 1.**
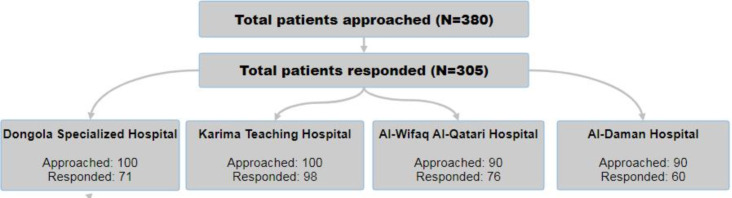
Flowchart illustrating the distribution of study participants across 4 participating hospitals in Northern State, Sudan.

A total of 380 patients undergoing elective surgery were approached, of whom 305 participated in this study (response rate=80.3%). Figure 1 shows the distribution of participants across the 4 participating hospitals. Approximately half of the participants were male (n=173, 56.7%) and aged between 31 and 65 years (n=161, 52.7%), with a median age of 43 (IQR 30‐64) years. Most participants (n=207, 67.9%) were married, and the majority (n=248, 81.3%) had some level of education. Of the 305 participants, 114 (37.4%) were freelancers, and 117 (38.4%) were unemployed. Nearly half of the participants (n=151, 49.5%) had an average monthly income above US $250. Family support was reported by 83.0% of participants (253/305). The sociodemographic characteristics of the participants are summarized in Table 1.

**Table 1.** Sociodemographic and medical/surgical characteristics of patients undergoing elective surgery at 4 participating hospitals in Northern State, Sudan, in 2024-2025 (N=305).

Characteristic	Participants, n (%)
Sex	
Male	173 (56.7)
Female	132 (43.3)
Age^a^ (years)	
18‐30	86 (28.2)
31‐50	102 (33.4)
51‐65	59 (19.3)
>65	58 (19.0)
Occupation	
Employed	46 (15.1)
Freelancer	114 (37.4)
Student	28 (9.2)
Unemployed	117 (38.4)
Marital status	
Married	207 (67.9)
Unmarried	98 (32.1)
Educational level	
Some level of education	248 (81.3)
No education	57 (18.7)
Average monthly income (US $)	
<50	125 (41.0)
50‐250	29 (9.5)
>250	151 (49.5)
Family support	
Yes	253 (83.0)
No	52 (17.0)
Type of hospital	
Public hospital	169 (55.4)
Private hospital	136 (44.6)
ASA^b^ classification	
ASA I and II	268 (87.9)
ASA III and IV	37 (12.1)
Type of surgery	
Minor	27 (8.9)
Intermediate	136 (44.6)
Major	142 (46.6)
Type of anesthesia	
General	160 (52.5)
Spinal	124 (40.7)
Local	21 (6.9)
Operation covered by insurance	
Yes	41 (13.4)
No	264 (86.6)
Subjective sleep quality the night before the operation	
Good	221 (72.5)
Poor	84 (27.5)
Previous operations	
Yes	119 (39.0)
No	186 (61.0)
Chronic diseases	
Yes	106 (34.8)
No	199 (65.2)

a Median age 43 (IQR 30-64) years.

b ASA: American Society of Anesthesiologists.

### Medical and Surgical Characteristics of the Study Population

The medical and surgical characteristics of the participants are detailed in Table 1. According to the American Society of Anesthesiologists (ASA) classification system, most patients (268/305, 87.9%) were classified as ASA I and II. Most patients underwent major (n=142, 46.6%) or intermediate (n=136, 44.6%) surgeries. General anesthesia was the most common type of anesthesia used (n=160, 52.5%), and most surgeries (n=169, 55.4%) were performed in public hospitals. Most surgeries (n=264, 86.6%) were not covered by insurance, and most patients (n=221, 72.5%) reported good subjective sleep quality the night before the operation. A significant proportion of patients had had no previous operations (n=186, 61.0%) and no chronic diseases (199, 65.2%).

### Prevalence of Preoperative Anxiety and Desire for Information

The prevalence of preoperative anxiety and desire for information among the participants is shown in Tables2  3. Overall, 75 of the 305 participants (24.6%) reported experiencing clinically significant preoperative anxiety. Of the 305 participants, 73 (23.9%) expressed an average desire for information, and 19 (6.2%) reported a high desire for information.

**Table 2.** Amsterdam Preoperative Anxiety and Information Scale (APAIS) scores of patients undergoing elective surgery at 4 participating hospitals in Northern State, Sudan, in 2024-2025 (N=305).

Statement	Not at all, n (%)	Slightly, n (%)	Moderately, n (%)	Quite a bit, n (%)	Extremely, n (%)
I am worried about the anesthesia	148 (48.5)	82 (26.9)	38 (12.5)	30 (9.8)	7 (2.3)
The anesthetic is on my mind continually	157 (54.5)	68 (22.3)	51 (16.7)	23 (7.5)	6 (2.0)
I would like to know as much as possible about the anesthesia	169 (55.4)	66 (21.6)	43 (14.1)	21 (6.9)	6 (2.0)
I am worried about the procedure	127 (41.6)	73 (23.9)	54 (17.7)	43 (14.1)	8 (2.6)
The procedure is on my mind continually	123 (40.3)	79 (25.9)	51 (16.7)	44 (14.4)	8 (2.6)
I would like to know as much as possible about the procedure	132 (43.3)	85 (27.9)	52 (17.0)	26 (8.5)	10 (3.3)

**Table 3.** Frequency of clinically significant preoperative anxiety and desire for information among patients undergoing elective surgery at 4 participating hospitals in Northern State, Sudan, in 2024-2025 (N=305).

Characteristic	Participants, n (%)
Clinically significant preoperative anxiety	
Present	75 (24.6)
Absent	230 (75.4)
Desire for information	
No desire	213 (69.8)
Average desire	73 (23.9)
High desire	19 (6.2)

### Factors Associated With Preoperative Anxiety and Desire for Information

The factors associated with preoperative anxiety (Table 4) included the presence of family support (*P*=.02), poor subjective sleep quality the night before the operation (*P*=.01), surgery in a public hospital (*P*<.001), ASA classification III and IV (*P*=.02), and a history of chronic diseases (*P*=.05).

**Table 4.** Association of sociodemographic and medical/surgical characteristics with clinically significant preoperative anxiety among patients undergoing elective surgery at 4 participating hospitals in Northern State, Sudan, in 2024-2025 (N=305).

Characteristic	Clinically significant preoperative anxiety			
	Present, n (%)	Absent, n (%)	Chi-square (*df*)	*P* value
Sex			0.90 (1)	.34
Male	39 (22.5)	134 (77.5)		
Female	36 (27.3)	96 (72.7)		
Age (years)			2.05 (3)	.56
18‐30	20 (23.3)	66 (76.7)		
31‐50	30 (29.4)	72 (70.6)		
51‐65	13 (22.0)	46 (78.0)		
>65	12 (20.7)	46 (79.3)		
Occupation			4.77 (3)	.19
Employed	13 (28.3)	33 (71.7)		
Freelancer	21 (18.4)	93 (81.6)		
Student	10 (35.7)	18 (64.3)		
Unemployed	31 (26.5)	86 (73.5)		
Marital status			0.29 (1)	.59
Married	49 (23.7)	158 (76.3)		
Single	26 (26.5)	72 (73.5)		
Educational level			1.88 (1)	.17
Some level of education	65 (26.2)	183 (73.8)		
No education	10 (17.5)	47 (82.5)		
Average monthly income (US $)			2.04 (2)	.36
<50	36 (28.8)	89 (71.2)		
50.00‐250	33 (21.9)	118 (78.1)		
>250	6 (20.7)	23 (79.3)		
Family support			5.76 (1)	.02
Yes	69 (27.3)	184 (72.7)		
No	6 (11.5)	46 (88.5)		
Type of hospital			17.01 (1)	<.001
Public hospital	57 (33.7)	112 (66.3)		
Private hospital	18 (13.2)	118 (86.8)		
ASA^a^ classification			5.78 (1)	.02
ASA I and II	60 (22.4)	208 (77.6)		
ASA III and IV	15 (40.5)	22 (59.5)		
Type of surgery			2.65 (2)	.27
Minor	9 (33.3)	18 (66.7)		
Intermediate	28 (20.6)	108 (79.4)		
Major	38 (26.8)	104 (73.2)		
Type of anesthesia			0.46 (2)	.79
General	37 (23.1)	123 (79.9)		
Spinal	32 (25.8)	92 (74.2)		
Local	6 (28.6)	15 (71.4)		
Operation covered by insurance			3.75 (1)	.11
Yes	6 (14.6)	35 (85.4)		
No	69 (26.1)	195 (73.9)		
Subjective sleep quality the night before the operation			7.74 (1)	.01
Good	45 (20.4)	176 (79.6)		
Poor	30 (35.7)	54 (64.3)		
Previous operations			1.35 (1)	.25
Yes	25 (21.0)	94 (79.0)		
No	50 (26.9)	136 (73.1)		
Chronic diseases			3.75 (1)	.05
Yes	33 (31.1)	73 (68.9)		
No	42 (21.1)	157 (78.9)		

a ASA: American Society of Anesthesiologists.

The factors associated with the desire for information (Table 5) included being employed (*P*=.01), being educated (*P*=.003), having family support (*P*=.03), undergoing surgery in a public hospital (*P*<.001), ASA classification III and IV (*P*=.001), and having chronic diseases (*P*=.02).

**Table 5.** Association of sociodemographic and medical/surgical characteristics with desire for information among patients undergoing elective surgery at 4 participating hospitals in Northern State, Sudan, in 2024-2025 (N=305).

Characteristic	Desire for information			
	No or little desire, n (%)	Average or high desire, n (%)	Chi-square (*df*)	*P* value
Sex			1.11 (1)	.29
Male	125 (72.3)	48 (27.7)		
Female	88 (66.7)	44 (33.3)		
Age (years)			4.06 (3)	.26
18‐30	63 (73.3)	23 (26.7)		
31‐50	65 (63.7)	37 (36.3)		
51‐65	40 (67.8)	19 (32.2)		
>65	45 (77.6)	13 (22.4)		
Occupation			12.22 (3)	.01
Employed	24 (52.2)	22 (47.8)		
Freelancer	90 (78.9)	24 (21.1)		
Student	21 (75.0)	7 (25.0)		
Unemployed	78 (66.7)	39 (33.3)		
Marital status			0.01 (1)	.91
Married	145 (70.0)	62 (30.0)		
Single	68 (69.4)	30 (30.6)		
Educational level			8.66 (1)	.003
Some level of education	164 (66.1)	84 (33.9)		
No education	49 (85.0)	8 (14.0)		
Average monthly income (US $)			0.71 (2)	.70
<50	88 (70.4)	37 (29.6)		
50‐250	103 (68.2)	48 (31.8)		
>250	22 (75.9)	7 (24.1)		
Family support			4.92 (1)	.03
Yes	170 (67.2)	83 (32.8)		
No	43 (82.7)	9 (17.3)		
Type of hospital			20.46 (1)	<.001
Public hospital	100 (59.2)	69 (40.8)		
Private hospital	113 (83.1)	23 (16.9)		
ASA^a^ classification			11.41 (1)	.001
ASA I and II	196 (73.1)	72 (26.9)		
ASA III and IV	17 (45.9)	20 (54.1)		
Type of surgery			1.14 (2)	.57
Minor	21 (77.8)	6 (22.2)		
Intermediate	92 (67.6)	44 (32.4)		
Major	100 (70.4)	42 (29.6)		
Type of anesthesia			0.44 (2)	.80
General	114 (71.3)	46 (28.8)		
Spinal	84 (67.7)	40 (32.3)		
Local	15 (71.4)	6 (28.6)		
Operation covered by insurance			1.79 (1)	.18
Yes	26 (63.4)	14 (34.1)		
No	187 (70.8)	59 (22.3)		
Subjective sleep quality of the night before the operation			0.22 (1)	.64
Good	156 (70.6)	65 (29.4)		
Poor	57 (67.9)	27 (32.1)		
Previous operations			0.55 (1)	.46
Yes	86 (72.3)	33 (27.7)		
No	127 (68.3)	59 (31.7)		
Chronic diseases			0.59 (1)	.02
Yes	65 (61.3)	41 (38.7)		
No	148 (74.4)	51 (25.6)		

a ASA: American Society of Anesthesiologists.

The results of the logistic regression analysis (Table 6) indicated that patients who had family support were 7 times more likely to develop preoperative anxiety (aOR 7.12, 95% CI 2.64‐19.23; *P*<.001). Patients treated in public hospitals had more than 4 times the risk of preoperative anxiety compared with those treated in private hospitals (aOR 4.31, 95% CI 2.30-8.07; *P*<.001). Patients with poor subjective sleep quality the night before the operation and those classified as ASA III and IV had more than 2 times the risk of preoperative anxiety compared with their counterparts (aOR 2.85, 95% CI 1.51‐5.38; *P*=.001; and aOR 2.36, 95% CI 1.00‐5.54; *P*=.049, respectively).

**Table 6.** Risk assessment of factors affecting clinically significant preoperative anxiety and desire for information, using binary and multinomial logistic regression (N=305).

Scale and variable	cOR^a^ (95% CI)	*P* value	aOR (95% CI)	*P* value
Preoperative anxiety				
Family support: yes (reference: no)	2.88 (1.18‐7.03)	.02	7.12 (2.64‐19.23)	<.001
Type of hospital: public (reference: private)	3.35 (1.85‐6.02)	<.001	4.31 (2.30‐8.07)	<.001
Subjective sleep quality the night before the operation: poor (reference: good)	2.17 (1.25‐3.78)	.01	2.85 (1.51‐5.38)	.001
ASA^b^ classification: ASA III/IV (reference: ASA I/II)	2.36 (1.16‐4.84)	.02	2.36 (1.00‐5.54)	.049
Chronic diseases: yes (reference: no)	1.69 (0.99‐2.88)	.05	1.14 (0.61‐2.14)	.61
Desire for information				
Occupation (reference: student)				
Freelancer	0.80 (0.30‐2.10)	.65	0.78 (0.29‐2.14)	.64
Employed	2.75 (0.98‐7.72)	.06	2.58 (0.89‐7.49)	.08
Unemployed	1.50 (0.59‐3.83)	.40	1.55 (0.57‐4.19)	.39
Educational level: some level of education (reference: no education)	3.14 (1.42‐6.93)	.01	2.48 (1.00‐6.11)	.049
Type of hospital: public (reference: private)	3.39 (1.97‐5.84)	<.001	3.57 (1.93‐6.61)	<.001
Family support: yes (reference: no)	2.33 (1.09‐5.01)	.03	4.10 (1.81‐9.30)	.001
ASA^b^ classification: ASA III/IV (reference: ASA I/II)	3.20 (1.59‐6.45)	<.001	3.26 (1.39‐7.64)	.007
Chronic diseases: yes (reference: no)	1.83 (1.11‐3.03)	.02	1.30 (0.72‐2.37)	.39

a cOR: crude odds ratio.

b ASA: American Society of Anesthesiologists.

Patients with some level of education were more than 2 times more likely to have a desire for information than patients with no education (aOR 2.48, 95% CI 1.00‐6.11; *P*=.049). Similarly, patients with family support were more than 4 times more likely to have a desire for information than those without family support (aOR 4.10, 95% CI 1.81‐9.30; *P*=.001). Patients treated in public hospitals had more than 3 times the desire for information compared with those treated in private hospitals (aOR 3.57, 95% CI 1.936.61; *P<*.001). Furthermore, ASA III and IV patients were more than 3 times more likely to have a desire for information than ASA I and II patients (aOR 3.26, 95% CI 1.39‐7.64; *P*=.007).

## Discussion

### Principal Findings

Preoperative anxiety is a common psychological response among patients awaiting surgery, with significant implications for their overall well-being, surgical outcomes, and recovery [22]. This study assessed the prevalence of preoperative anxiety and desire for information among patients scheduled for elective surgery in Northern State, Sudan, and identified the factors associated with these outcomes. A summary of what is known about this topic and what this study adds is shown in Textbox 1.

Textbox 1. **What Is Already Known on This Topic**

- Preoperative anxiety is a common issue affecting surgical outcomes and patient well-being, with varying prevalence rates worldwide.
- Patients often desire more information than they receive, which may impact their satisfaction and anxiety levels.
- Previous studies have identified that factors such as sex, educational level, and American Society of Anesthesiologists (ASA) classification influence preoperative anxiety.

**What This Study Adds**

- Public and private health care settings may have different impacts on patient anxiety and information needs due to resource availability and patient experience.
- Certain cultural factors, such as extensive family involvement, may paradoxically amplify preoperative anxiety in certain regions.
- Educated patients, patients with higher ASA classification, and patients being treated in public hospitals are more likely to seek information.

### Comparison With Prior Work

The study found that 24.6% of participants experienced preoperative anxiety, a rate lower than the global prevalence and that observed in LMICs (48% and 55.7%, respectively) [6,7]. However, studies from Yemen, Nigeria, Palestine, India, Nepal, and France are consistent with our results, reporting rates of 29.8%, 24.4%, 27.1%, 21.0%, 25.8%, and 24.7%, respectively [9,23-27]. These variations may be attributed to diverse factors, such as differences in health care systems, geographical locations, study populations, and assessment tools used.

Our study identified several factors linked to preoperative anxiety, including family support, poor subjective sleep quality, surgery in a public hospital, and ASA classification III and IV. Remarkably, patients with family support were 7 times more likely to experience preoperative anxiety (aOR 7.12, 95% CI 2.64‐19.23; *P*<.001), which contrasts with the conventional view of family support as a protective factor. The unexpected result may stem from unique family dynamics in Sudan, where extended visits from relatives and community members expressing concerns or sharing negative experiences may inadvertently heighten a patient’s anxiety. This underscores the complex role of social support in preoperative anxiety. Previous studies have reported conflicting results, with some suggesting that family support reduces anxiety and others indicating no significant impact [28,29].

Consistent with previous research, our study found that patients treated in public hospitals had a significantly higher risk of preoperative anxiety compared to those in private hospitals (aOR 4.31, 95% CI 2.30‐8.07; *P*<.001). A similar trend was observed in a study from Tanzania, which also reported higher preoperative anxiety among patients in public hospitals [30]. This disparity may be due to factors such as differences in resource availability, staffing levels, and the overall patient experience between public and private health care settings.

Poor sleep quality the night before surgery was significantly associated with preoperative anxiety, consistent with previous studies highlighting the strong link between sleep disturbances and anxiety disorders [31-34]. The stress and anticipation of surgery may disrupt sleep, exacerbating anxiety levels. Similarly, patients classified as ASA III and IV had a higher risk of preoperative anxiety, likely due to their increased risk of complications and poorer health status [35]. This finding aligns with several studies demonstrating a significant association between higher ASA classification and elevated anxiety levels [23,36,37].

Notably, 23.9% of the patients expressed an average desire for information, and 6.2% reported a high desire for information. This desire, as part of broader health information–seeking behavior, enhances health literacy. However, surgeons often underestimate the extent of information patients wish to receive preoperatively [38,39]. Similar patterns have been observed in studies from Yemen and Nigeria, where comparable percentages of patients expressed average and high desire for information (16.5% and 16.9% in Yemen, and 14.4% and 5.1% in Nigeria, respectively) [9,23].

Our study reveals that patients with some education are more likely to seek information. One possible explanation is that more educated patients may feel more confident in asking questions and navigating medical discussions, whereas those with less formal education may feel less empowered or perceive themselves as less able to understand complex medical explanations. Patients in public hospitals also showed a higher desire for information, possibly due to perceived communication gaps. Additionally, ASA III and IV patients had a stronger desire for information, likely driven by the need to understand their health risks. To the best of our knowledge, this study is the first to comprehensively explore these factors, addressing a significant gap in the literature and offering insights for enhancing patient-centered care.

### Conclusion

This study found that a notable proportion of surgical patients in Northern State, Sudan, experienced preoperative anxiety, a rate lower than the global average but consistent with some local and international findings. Factors associated with increased anxiety included family support, poor sleep quality, public hospital settings, and higher ASA classification. The study also highlighted a significant desire for information among patients, particularly those with some education and those receiving treatment in public hospitals. Addressing these factors through targeted interventions may improve patient outcomes and satisfaction.

### Recommendations

Several recommendations can be made on the basis of these study findings to enhance patient care and reduce preoperative anxiety. Health care providers should implement targeted interventions aimed at improving preoperative sleep quality and providing adequate information to patients, as these factors were significantly linked to anxiety levels. Additionally, while family visits may help reduce preoperative anxiety for some patients, they may contribute to increased anxiety in certain situations. Clinical teams should assess the potential risks and benefits of family visits in the preoperative period and act accordingly. Furthermore, public hospitals should prioritize improving patient communication and addressing perceived gaps in information provision, as these factors were associated with a higher desire for information among patients.

### Limitations and Strengths

This study has several limitations. The small sample size and the use of convenience sampling may limit generalizability, but the multicenter study design enhances representativeness. The cross-sectional design prevents establishing causality; however, it provides a detailed snapshot for future longitudinal studies. The reliance on self-reported data introduces potential biases, but using a validated assessment tool and face-to-face interviews helped minimize these issues.

## Supplementary material

10.2196/75736 Multimedia Appendix 1 English version of the questionnaire.

## Abbreviations

aOR: adjusted odds ratio
APAIS: Amsterdam Preoperative Anxiety and Information Scale
ASA: American Society of Anesthesiologists
LMICs: low- and middle-income countries
STROBE: Strengthening the Reporting of Observational Studies in Epidemiology
STROBE: Strengthening the Reporting of Observational Studies in Epidemiology
