# Supplementary material for: Preoperative Anxiety and Information Desire Among Patients Undergoing Elective Surgery in Northern Sudan: Multicenter Cross-Sectional Study
Source: JMIR Perioper Med. 2025 Oct 15;8:e75736. doi: 10.2196/75736 (PMC12526657; doi:10.2196/75736)
Supplement: Multimedia Appendix 1 [file periop-v8-e75736-s001.docx]

Questionnaire

Note:

- Please take your time and answer the following interview questions honestly and thoughtfully.
- Your responses are important and will be kept strictly confidential. There are no right or wrong answers—what matters most is your personal experience and opinion.
- If you feel unsure about a question, feel free to ask for clarification.
- Participation is voluntary, and you may skip any question or withdraw at any time without any consequences.

**Serial Number: _____________________**

# Socio-demographic Data

1. **Sex:**

Male [ ] Female [ ]

1. **Age:** ________________ (in years)
2. **Occupation:**

Employed [ ] Freelance [ ] Student [ ] Unemployed [ ]

1. **Marital Status**:

Married [ ] Unmarried [ ]

1. **Educational Level:**

No Formal Education [ ] Primary [ ] Secondary [ ] University [ ]

1. **Average Monthly Income (in USD):**

<50.00$ [ ] 50.00 – 250.00$ [ ] >250.00$Education [ ]

1. **Family Support:**

Yes [ ] No [ ]

# Medical and Surgical History

1. **Type of Hospital:**

Public [ ] Private [ ]

1. **ASA Classification:**

ASA classes I and II [ ] ASA classes III and IV [ ]

1. **Type of Surgery:**

Minor [ ] Intermediate [ ] Major [ ]

1. **Type of Anesthesia:**

General [ ] Spinal [ ] Local [ ]

1. **Operation Covered by Insurance:**

Yes [ ] No [ ]

1. **Subjective Sleep Quality Before Operation:**

Good [ ] Poor [ ]

1. **Previous Operations:**

Yes [ ] No [ ]

1. **Chronic Diseases:**

Yes [ ] No [ ]

# Assessment of Preoperative Anxiety and Desire for Information using the The Amsterdam Preoperative Anxiety and Information Scale (APAIS)

- Please tick (✔) the appropriate cell that best reflects your opinion for each statement below.
- There are no right or wrong answers—choose the option that most closely matches how you feel.

| **Question** | **NAA** | **SLT** | **MOD** | **QAB** | **EXT** |
| --- | --- | --- | --- | --- | --- |
| 1. I am worried about the anesthesia |  |  |  |  |  |
| 1. The anesthetic is on my mind continually |  |  |  |  |  |
| 1. I would like to know as much as possible about the anesthesia |  |  |  |  |  |
| 1. I am worried about the procedure |  |  |  |  |  |
| 1. The procedures on my mind continually |  |  |  |  |  |
| 1. I would like to know as much as possible about the procedure |  |  |  |  |  |

**Keys:** NAA = Not at all; SLT = Slightly; MOD = Moderately; QAB = Quite a bit; EXT = Extremely
